# Optimizing Nutrient Dynamics for Crop Resilience to Abiotic Stress: An Endogenous Phytohormone Perspective

**DOI:** 10.3390/plants14213303

**Published:** 2025-10-29

**Authors:** Ibragim Bamatov, Eliza Sobralieva, Rashiya Bekmurzaeva, Shamil Alimurzaev

**Affiliations:** 1 All-Russian Research Institute of Reclaimed Lands, V. V. Dokuchaev Soil Science Institute, Moscow 119017, Russia; 2 Department of Horticulture and Viticulture, Kadyrov Chechen State University, Grozny 364024, Russia; elissobr@inbox.ru (E.S.); shamil-alimurzaev@mail.ru (S.A.); 3 Department of Ecology and Nature Management, Kadyrov Chechen State University, Grozny 364024, Russia; raya.bek@yandex.ru

**Keywords:** phytohormone, nutrient deficiency, signal transduction, omics approach, cross-talk

## Abstract

Plants continuously adapt to dynamic environmental conditions, which include abiotic stress such as drought, salinity, and high temperature. Translocation, availability, and uptake of essential nutrients are suggested to be disrupted, thereby impairing growth, development, and productivity of the plant. The interplay between the root architecture, membrane transporters, and hormonal regulation is suggested to have efficient nutrient acquisition. For mediating nutrient uptake and redistribution under abiotic stress conditions, transporter proteins such as nitrate (NRT), ammonium (AMT), phosphate (PHT), and potassium (HAK) families play a crucial role for the major essential elements (N, P, K). Abiotic stress triggers specific transcriptional and post-transcriptional regulation of these transporters, modulating their activity in response to external nutrient availability. Under nutrient-deficient conditions, phytohormones such as abscisic acid (ABA), cytokinin, and ethylene play a pivotal role in orchestrating plant responses. Moreover, the plant stress tolerance is suggested to be influenced by stress-induced signalling mechanisms, which are mediated by reactive oxygen species (ROS). The current review synthesizes current knowledge of nutrient dynamics under abiotic stress, focusing on the molecular mechanisms governing transporter regulation and phytohormonal crosstalk. By unravelling these complex regulatory networks, this article aims to pave the way for sustainable agricultural practices.

## 1. Introduction

The current trend of population growth is expected to reach 9–10 billion by 2050, posing an unprecedented challenge to global food security [1]. Meeting this escalating demand necessitates an increase in crop productivity along with the development of sustainable agricultural practices that minimize environmental impact [2]. The new trend in green revolution farming relies heavily on using fertilizers and pesticides, which are expensive if valued in economic and environmental terms [3]. Hence, it would be important to understand in a wider depth the way plants respond to the lack of minerals so that the availability of nutrients can shape the vulnerability in the field to different stresses. The imperative, therefore, is to foster the development of crops that exhibit the enhanced nutrient use efficiency (NUE) that enables them to absorb and utilize the available nutrients more effectively [4]. The above goal can be achieved by understanding how the plant perceives, acquires, transports, utilizes, and changes the dynamics of mineral nutrients and how they adapt to cope with the nutrient-deficient or toxic conditions.

Plants have evolved significantly with adaptive mechanisms to acclimate and thrive under the diverse and often challenging environmental conditions, particularly in relation to nutrient availability [5]. One of the primary strategies involves modifying their root system architecture (RSA). Through root plasticity, plants adjust the branching patterns and overall structure of their roots to enhance nutrient uptake under nutrient-deficient conditions [6]. Plant response to the different deficiencies of macronutrients such as nitrogen, phosphorus, and potassium involves modifying their root system architecture. Nitrogen is the most crucial macronutrient, which is required for plant growth and development, as it is required in larger quantities and is also involved as a building block of amino acids [7]. The nitrogen is absorbed as nitrate (NO_3_^−^) and ammonium (NH_4_^+^) in plant; however, amino acids and other organic nitrogen compounds can also contribute to nitrogen uptake in plants [8]. Nitrogen uptake in plants is mediated by ammonium transporters (AMTs) and nitrate transporters (NRTs). In rice, the OsAMT1 family are plasma membrane proteins responsible for NH_4_^+^ uptake by root cells. Moreover, *OsAMT1* genes show tissue-specific expression and regulate ammonium absorption [9]. Similarly, NRT1 and NRT2 families of protein mediate NO_3_^−^ uptake and translocation in plants. For example, *AtNRT2*.1 and *AtNRT2*.2 are high-affinity nitrate transporters in *Arabidopsis*, and their disruption can severely impair NO_3_^−^ uptake and plant growth [4,10]. In regard to phosphorus starvation, the phosphorus starvation tolerance 1 (*PSTOL1*) gene has been identified as the crucial determinant in providing tolerance to low-phosphorus conditions in rice and also plays an important role in improving root system architecture and yield [11]. Similarly, the gene line *TRH1* also plays a crucial role in regulating root hair elongation, which encodes for a potassium transporter belonging to the *AtKT/AtKUP/HAK* family [12]. Potassium is a key nutrient that helps in plant growth, stress tolerance, and defence mechanisms. In plants, K^+^ uptake from the soil and its subsequent transport and distribution within plant cells are primarily facilitated by K^+^ channels and transporters, including the HAK/KUP/KT (High-affinity K^+^ transporter/K^+^ uptake permease/K^+^ transporter) family, which represents the largest group of K^+^ transporters in plants [13]. K deficiency triggers metabolic adjustments such as oxylipin and glucosinolate production, linking nutrient status with plant defence and secondary metabolism [14].

The nutrient dynamics in plant stress tolerance are mainly regulated by the involvement of phytohormones. Phytohormones mediate adaptive changes in plant root architecture, which are essential for nutrient acquisition from the soil, primarily when nutrients are sparsely distributed or of limited solubility [15]. For instance, auxin (IAA), is a key regulator responsible for mediating plant response to localized nitrate availability and influencing lateral root initiation [16]. On the contrary, phytohormones such as abscisic acid (ABA) are involved in the nitrate inhibitory effect on primary root growth with transporters like MtNPF6.8 and MtNPF1.7, which act in ABA-dependent nitrate signalling [17]. On the other hand, cytokinin regulates the root development and nitrate induction of primary root growth [18]. Jasmonic acid is also involved in plant defence against herbivores, which is induced under potassium deficiency and also can suppress the iron deficiency gene expression [14,19]. Furthermore, hormones such as ethylene interact with auxin to modulate root formation [20]. The interplay between these hormones and nutrient signalling is complex, involving extensive crosstalk, as seen in how deficiencies in nitrogen, phosphorus, and potassium can affect the expression of microRNAs (miRNAs) that, in turn, regulate nutrient responses [21,22]. This review aims to provide an overview of recent advances in understanding the mechanisms of plant nutrient uptake, transport, and homeostasis, with a particular focus on the molecular and physiological basis of these processes and their intricate regulation by phytohormones, offering insights for future crop engineering towards a more sustainable and productive agriculture.

## 2. Nutrient Dynamics in Plants Under Abiotic Stress

### 2.1. Nutrient Mobilization in Soil and Response to Abiotic Stress

Nutrient mobilization in the soil depends upon many biotic and abiotic factors that modulate nutrient uptake in the plant [23]. The growth and development of plants might be affected by variations in the nutrient uptake by the plant and its genetic factors. While significant progress has been made, the complex interplay of physiological and biochemical properties within the rhizosphere and their precise regulation under combined stresses require further elucidation, which further complicates the understanding of nutrient acquisition from soil. However, plants adapt themselves in the soil and make the root system architecture inside the soil, which promotes weathering of soil, mobilization of nutrients and, ultimately, uptake of nutrients via the root [6]. Another mechanism in the plant where the uptake efficiency from the root was reported to be enhanced is the synthesis of root exudates [24]. These processes enhance the uptake of minerals from the rhizosphere to root tissues [23]. These aforementioned processes in the rhizosphere lead to nutrient uptake from soil to the plant system. The root provides resilience against abiotic stress, and the properties of root system architecture must be evaluated to comprehend the nutrient uptake in the plant, which ultimately supplies the necessary for plant growth and development [25]. A lack of mineral nutrients and various environmental restrictions (including abiotic and biotic stress) are frequent causes of low crop production and productivity. Nutrient imbalances substantially affect plant performance, including growth patterns, antioxidant defence systems, and tolerance to biotic and abiotic stresses [25]. Under various environmental conditions, low productivity is common due to a lack of mineral nutrient supply. Micronutrient disorders are common nutritional imbalances in plants that significantly impact plant performance and response to their surroundings [26]. Micronutrient deficits have secondary, often unintended impacts on plant growth via alterations in growth pattern, chemical composition, and antioxidant defence capability and, in particular, diminish plant resilience to biotic and abiotic environmental challenges [25].

**Figure 1:**
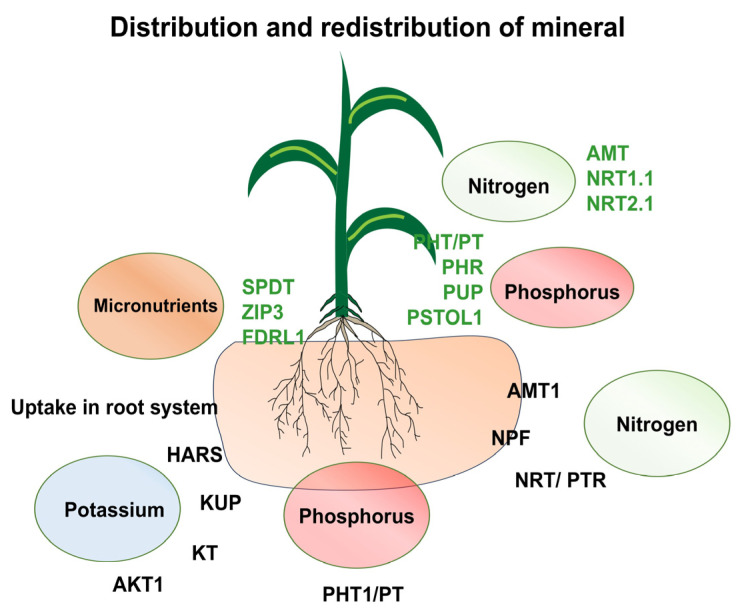
A model illustrating tissue-specific expression patterns of key nutrient transporters such as nitrogen (NRT, NPF, AMT1), phosphorus (PHT1, PT), and potassium (HARS, KUP, KT, AKT1) in the root. Similarly, in shoots the uptake of micro and macronutrients takes place via transporters of nitrogen (AMT, NRT1.1, NRT2.1), phosphorus (PHT/PT, PHR, PUP, PSTOL1), and micronutrients (SPDT, ZIP3, FDRL1). Abiotic stress is known to trigger specific transcriptional and post-transcriptional regulation of these transporters, modulating their activity in response to external nutrient availability.

Plants are constantly subjected to dynamic and often challenging environmental conditions, particularly various abiotic stresses such as drought, salinity, and temperature extremes [27]. A significant consequence of these environmental pressures is the disruption of the availability, uptake, and movement of essential nutrients, which consequently impairs plant growth, development, and overall [28,29]. Efficient nutrient acquisition and assimilation are complex processes, relying on the intricate interplay of root architecture, membrane transporters, and sophisticated hormonal regulation (Figure 1). Variations in nutrient uptake, influenced by genetic factors and both biotic and abiotic elements, can profoundly affect plant growth and development [6,30,31]. In response to fluctuating and often nutrient-deficient conditions in the rhizosphere, plants employ adaptive strategies involving modulations of their root system architecture and morphophysiological aspects [32]. These adjustments include altering the root’s total surface area and overall structure. For example, under limiting conditions of nitrogen, sulphur, or phosphorus, plants may modify their lateral root structure to increase the surface area available for nutrient absorption. Specifically, under low-phosphorus conditions, plants exhibit changes in root morphology, such as an increase in the number and length of root hairs, which expands the root surface [33]. This restructuring of the root system is a crucial adaptive mechanism for efficient phosphorus acquisition. These root modifications facilitate the proper allocation of photoassimilates from source organs to sink organs, ultimately contributing to root development and resulting in enhanced root-to-shoot ratios in nutrient-limited plants [34].

### 2.2. Nutrient Uptake by Root and Involvement of Various Transporters Under Abiotic Stress

The major targets for enhancing the efficiency of water uptake and transfer of nutrients are the proteins that are associated with the transporter present on the plasma membrane (MTs) [35]. The MTs induce cellular homeostasis and play a major role in regulating ionic fluxes through cellular channels from roots to other parts of the plant. The other activities that are involved are xylem loading and transport of sugar molecules from the source (photosynthetic tissues in the leaf) to sink tissues (roots, stem, and seeds) [36]. Membrane transporters are essential for plant growth and development in terms of increased plant height, branches/tillers, better quantity, length, and filled panicles per plant, seed yield, and grain quality [35]. Due to poor selectivity, some membrane transporters uptake toxic elements in roots, which negatively impact plant growth and development and are later transferred to sink tissue such as grain, where they degrade grain quality [28].

In arid and semi-arid conditions, plant growth and agricultural productivity are severely constrained by soil water availability. Constant drought stress can lead plants to develop reactive oxygen species (ROS), damaging leaves and eventually limiting crop yield [37]. Nitrogen (N) is an essential element growth and development of the plant, and its productivity also depends upon the exogenous N application in soil. Generally, soil contains two types of inorganic N: ammonium ions (NH_4_^+^) and nitrate ions (NO_3_^–^) [38]. In cereal crops such as rice, which is typically grown in flooded soil conditions, where NH_4_^+^ is the major type of nitrogen that plants uptake from the root. Rice has two uptake systems for ammonium ion (NH_4_^+^). Firstly, according to physiological analysis of the uptake of nitrogen: a high-affinity transport system (HATS) and a low-affinity transport system (LATS) [39]. Similarly, the development of the root and variation in the diffusion rate of K^+^ in the soil were reported to be restricted under drought conditions, thereby inhibiting the K uptake in the plant. Lower K concentrations can further reduce drought tolerance and K absorption in plants. Maintaining enough K levels is, therefore, essential for plant drought resistance. It has been demonstrated that K nutritional status and plant drought resistance are closely related [40].

In *Arabidopsis*, the first transporter of nitrogen was discovered, i.e., the ammonium transporter gene (*AMT1*), which was reported to be involved in the HATS [41]. It was suggested that about 12 putative AMT members of the transporter class were involved in rice. However, only a few of them have been defined functionally according to their localization, expression pattern, and transport activity in the plant [42]. Reports have suggested that the AMT transporters in rice, *OsAMT1;1* and *OsAMT1;2,* are expressed preferentially in the roots, whereas *OsAMT2;2* is expressed equally in both roots and shoots [43]. The expression patterns of *SlAMT1* genes revealed that they were differentially expressed in response to stressors of drought and salt. When the expression of *SlAMT1* genes was studied in response to abiotic stressors, it was discovered that the expression in leaves and roots was predominantly downregulated [44]. *OsAMT1*;*2* mRNA is detected by in situ hybridization in the exodermis, sclerenchyma, and endodermis of the main root tip exodermis, sclerenchyma, and pericycle cells [9]. As a result, it was concluded that *OsAMT1;2* might be involved in the xylem loading and NH_4_^+^ absorption. Based on the overexpression studies, it was revealed that two more members of this family, named *OsAMT1*;*1* and *OsAMT1*;*3,* were also involved in the absorption of NH_4_^+^ [45]. Plant nitrate transporters (NPFs) are members of the NRT1/PTR (NITRATE TRANSPORTER 1/PEPTIDE TRANSPORTER) family protein. Numerous substrates, including nitrate and peptides, are transported by membrane transporters encoded by the NPF genes [46]. Other families of transporters, such as *OsNPF2*.2, were reported to be found on the cell membrane in rice, which is directly linked with the nitrate transport from root to shoot [47]. Reports have suggested that *OsNPF2*.2 is a nitrate-inducible, pH-dependent, low-affinity nitrate transporter predominantly located in the parenchyma cells surrounding the xylem. The disruption of *OsNPF2*.2 has increased nitrate content in shoot xylem exudates [47].

Phosphorus in the soil is majorly available in two forms of inorganic P (Pi), H_2_PO_4_^−^ and HPO_2_^4−^, depending upon the soil pH. Plasma membrane-localized Pi transporters facilitate Pi absorption with the help of the phosphate transporter (*PHT1/PT*) family. Studies have suggested that about thirteen *PHT1* genes in rice have been discovered, which regulate P homeostasis [48], and many of the P transporters are involved in Pi uptake from soil. *OsPht1;4 (PT4)* and *OsPht1;8 (PT8)* transporters are specifically expressed in roots, and their transporter activity has been validated in yeast or oocytes [49]. Other types of P transporters such as *OsPT2* are only present and expressed in the stele of primary and lateral roots, whereas OsPT6 is present in both the cortical and epidermal cells. However, the proportional contribution of *OsPT2* and *OsPT6* to total Pi uptake is unknown. On the other hand, analysis of collinearity studies revealed that the majority of *BnaPHT1* (Brassica napus *PHT1*) genes shared syntenic relationships with *PHT1* members in *Arabidopsis thaliana*, *Brassica rapa*, and *Brassica oleracea*, and that whole-genome duplication (polyploidy) played a major role in *BnaPHT1* evolution, in addition to segmental duplication. In response to phosphorus (P) shortage, transcript abundance analysis revealed that individual *BnaPHT1* genes exhibited diverse expression patterns. In addition, nutritional stressors such as nitrogen (N), potassium (K), sulphur (S), and iron (Fe) can modulate the expression levels of *BnaPHT1* genes. In addition, abiotic stress conditions, such as salt and drought conditions, can modulate the transcript abundances of *BnaPHT1s* and phytohormones, such as auxin and cytokinin [50].

Potassium (K) plays an important role in regulating osmotic pressure in plants. Under abiotic stress, the role of K increases manyfold for maintaining ionic homeostasis [14,22]. In *Arabidopsis*, the K uptake transporters were identified earlier. Two transporters have been reported to be associated with K^+^ uptake: *HAK5* (potassium transporter KUP/HAK/KT family) and *AKT1* (ARABIDOPSIS K^+^ TRANSPORTER1; shaker family potassium channel) [51]. The HAK/KUP/KT (High-affinity K^+^ transporter/K^+^ uptake permease/K^+^ transporter) family is the biggest potassium transporter family in plants and is essential for K^+^ uptake, transport, and biotic and abiotic stress responses [13]. Recent reports in barley suggested that 27 *HAK* genes (*HvHAKs*) have been discovered, and their tissue expression patterns and responses to salt stress, drought stress, and potassium deprivation are distinct [13]. The sensitivity of peach seedlings to polyethylene glycol (PEG), lead, and cadmium was indicated by stunted development, K^+^ deficiency, and poor photosynthetic efficiency. However, peach seedlings were aluminum-tolerant. K^+^ deficit enhanced *PpeKUP* gene expression in roots, whereas K^+^ excess decreased it. Al treatments increased *PpeKUP* transcription in shoots, while PEG, Pd, and Cd treatments increased *PpeKUP* transcription in all tissues [52]. However, transporters involved in K^+^ uptake in rice are poorly understood to date and need further investigation. A homolog of Arabidopsis *AKT1*, rice *AKT1* contributes to K^+^ uptake and functions as an inward-rectifying channel in the plant [53]. Low-K^+^ conditions have no effect on the level of *OsAKT1* expression in roots or shoots. In the presence of limited or normal K^+^ concentrations in the soil solution, the absence of *OsAKT1* decreased the biomass and enhanced K^+^ concentration of the roots and shoots during both vegetative and reproductive growth stages [53].

### 2.3. Nutrient Transport from Root to Leaves and Its Response to Abiotic Stress

The transport of nutrients from roots to leaves requires various transporters that are related to the partitioning of nutrients in the source and sink [54]. NRT2 proteins are part of a high-affinity transport (HAT) mechanism responsible for the transfer of nitrate at low concentrations in nitrogen-limited conditions. [4]. Out of seven, four *Arabidopsis* NRT2 transporters have been implicated in low nitrogen adaptation to date. These four transporters have distinct spatiotemporal distributions in the root, and the expression level changes under stress conditions [55]. Another form present in Arabidopsis, *AtNRT2.1*, was reported to be localized in mature root cortex cells and repressed by nitrogen-deficient conditions. The expression of *AtNRT2.1* mediates apoplastic nitrate absorption, the IHATS (nitrate-inducible high-affinity transport system), and root system architecture under abiotic stress such as low-nitrogen conditions [10].

Another study under nitrogen-limited conditions with nrt2.1, nrt2.2, and nrt2.4 triple mutants and nrt2.1 and nrt2.2 double mutants suggested that *AtNRT2.4* substantially contributed to enhancing the plant biomass under nitrate-deficient conditions. In addition, researchers also revealed that *AtNRT2.5*, along with *AtNRT2.1*, *AtNRT2.2*, and *AtNRT2.4*, was essential for *Arabidopsis* plants to endure severe nitrogen deficiency [4]. Similarly, *AtAMT1;1* from Arabidopsis is the first ammonium (NH_4_^+^) transporter discovered. It is responsible for transporting NH_4_^+^ ions and their partitioning throughout the plant [41]. Since then, *AtAMT1;2*, *AtAMT1;3*, *AtAMT1;4*, and *AtAMT1;5* have been identified as NH_4_^+^ transporters, which were reported to be localized on root cells and responsible for apoplastic and symplastic uptake from soil, and are high-affinity in nature [56]. In addition, Arabidopsis possesses an NH_4_^+^ transporter of the MEP type; *AtAMT2;1*. Under nitrogen stress conditions, four AMTs, including AtAMT1;1, AtAMT1;2, AtAMT1;3, and AtAMT2;1, were suggested to be overexpressed [56]. Expression of OsAMT1;1 and OsAMT1;2 in *Oryza sativa* is reported to be upregulated in the presence of high-nitrogen sources such as NH_4_^+^ ions, whereas OsAMT1;3 is significantly upregulated under nitrogen-deficient conditions [45]. The chloroplast uses the glutamine synthetase and glutamate synthase (GS-GOGAT) pathway to absorb the NH_4_^+^ ions generated during metabolic activities, including photorespiration, amino acid recycling, and decreased nitrate transport [56]. Although nitrate absorption often becomes predominant in roots under stress circumstances, such as low light intensity and restricted external nitrate supply, nitrate taken up by plants is normally transported over vast distances to aerial regions for further assimilation. This perplexing phenomenon lowers energy efficiency, which is not a behavior that supports plant survival. Thus, although hardly being understood, the underlying physiological importance and regulating processes continue to draw in many scientists [57].

### 2.4. Role of the Transporter and Genes in the Accumulation and Assimilation of Nutrients in the Sink Tissue

The accumulation and assimilation of nutrients in various tissues in the plant depend upon the transporter and demand of the tissue [52]. In graminaceous plants, such as rice, the node structure is a complex, well-organized vascular system that plays a crucial role in distributing and allocating different mineral elements. The transporter present in some nodes participates in ion distribution by facilitating intervascular transfer in the nodes [58]. In rice, transporters present in the upper node, such as *OsFRDL1,* which is a citrate efflux transporter, are involved in Fe solubilization and Fe deposition in the apoplastic portion [59]. Expression of the SULTR-like phosphorus distribution transporter (SPDT) in the node, which is plasma-membrane-localized phosphorus (Pi) transporter, mediates the distribution of Pi to the rice grains. Any mutation in the transporter gene decreases grain P while simultaneously increasing leaf P [60]. The knockout of the SPDT gene in rice showed decreased Pi accumulation in the grains but elevated Pi levels in the leaves of spd mutants [61]. *OsZIP3* acts as a zinc transporter in the node and regulates zinc distribution in the developing tissue of rice. This gene was silenced in the RNAi plant, resulting in lower zinc concentrations in the shoot meristem and elongation zone, but enhanced zinc accumulation in the mature leaves. However, the plant was unaffected by the root-to-shoot translocation [62].

Phosphate transporters (*PHT/PT*) in plants are located in the plasma membrane and tonoplast membrane and regulate phosphate uptake from the soil. In *Arabidopsis*, there are 22 members of the *PHT* family, which are distributed among five groups, viz., the *PHT2*, *PHT3*, *PHT4*, and *PHT5* gene families [63]. Reducing the expression of the phosphorus transporter genes *TaPht1;4* and *OsPht8* in wheat and rice plant roots, respectively, reduces phosphorus uptake [64,65]. Under P-deficient conditions, a set of genes crucial for maintaining phosphorus homeostasis within the plant is activated during phosphorus shortage. Overexpression of the phosphate-starvation-induced (IPS) gene Phosphate Starvation Response Regulator 2 (OsPHR2) in rice improves phosphorus levels in shoots by favorably influencing the expression of Phosphate Transporter 2 (also known as OsPht1;2) [65]. Phosphorus uptake1 (Pup1) is a QTL that encodes a protein kinase and is associated with high Pi uptake. It was reported for the first time in an aus-type rice cultivar, which was reportedly grown under phosphorus-deficient conditions in the northeastern region of India. Moreover, the yield of rice crops is also highly dependent upon Pup1 under P-deficient conditions, favored by the growth of root tissue [33]. Gamuyao et al. [11] have renamed Pup1 as phosphorus starvation tolerance 1 because of its sole occurrence in phosphorus-starvation-tolerant rice cultivars (PSTOL1). Additionally, the overexpression of PSTOL1 in low-phosphorus-sensitive rice cultivars boosts grain production in low-phosphorus soil. Positive control of root development by PSTOL1 increases Pi absorption even under situations of low Pi availability [11,66].

The absorption and assimilation of nitrogen (N) are significantly impacted by various abiotic stresses, including salt, drought, and high temperatures. These stresses can cause osmotic stress in plant cells and affect nutrient concentrations, particularly N, within the plant. For instance, N deficiency can hinder cell division and expansion, especially under drought, leading to reduced leaf production and development [67]. Proteins, such as those in the NRT1 and NRT2 families, are crucial for nitrate uptake and redistribution. NRT1.1 is a dual-affinity transporter and a nitrate sensor, playing a role in both low and high nitrate concentrations. NRT2 proteins are involved in high-affinity transport, particularly under nitrogen-limited conditions [10]. In Arabidopsis, NRT2.1, NRT2.2, NRT2.4, and NRT2.5 function under nitrate starvation, with NRT2.1 and NRT2.2 being major contributors to nitrate uptake under a limited N supply. NRT1.5 facilitates root-to-shoot nitrate transport by loading nitrate into the xylem. NRT1.7 mediates phloem loading of nitrate from older leaves to N-demanding tissues for remobilization. The plasma membrane proteins are responsible for transporting ammonium (NH_4_^+^) into plants. Examples include AMT1 in Arabidopsis and OsAMT1;1, OsAMT1;2, and OsAMT1;3 in rice. OsAMT1;2, detected in the exodermis, sclerenchyma, and endodermis of main root tips, may be involved in xylem loading and NH4+ uptake. AMT1.1 and AMT1.3, along with AMT1.5, are involved in direct soil uptake via the epidermis, while AMT1.2 is expressed in cortical and endodermal cells, mediating apoplastic absorption of ammonium [43,68]. Reports have suggested that GS-overexpressed rice plants, which had an elevated metabolic level, exhibited greater total GS activities and soluble protein concentrations in leaves, as well as greater total amino acids and total nitrogen content in the entire plant. In comparison to wild-type plants, GS-overexpressed plants produced less grain and had fewer total amino acids in their seeds. In addition, GS1;2-overexpressed plants displayed resistance to Basta selection and greater sensitivity to salt, drought, and cold stress conditions compared to wild-type plants, whereas the other two kinds of GS-overexpressed plants demonstrated no significant differences under these stress conditions [69].

## 3. Phytohormone-Mediated Regulation of Nutrient Transport and Assimilation in Plants

### 3.1. Importance of Phytohormone in Nutrient Uptake

Several abiotic stresses, including drought, extreme temperature, and salinity, impose pressure on plants. Abiotic stresses negatively influence the physiology and morphology of plants by disrupting the genetic regulation of cellular activities (Table 1). Phytohormones are among the most important growth regulators; they exert a considerable effect on plant metabolism and play a critical role in the activation of plant defence systems in response to abiotic stresses. Under abiotic stress conditions, the external application of the phytohormone could promote plant development and metabolism [70]. Nutrients have limited solubility and are unevenly distributed in the soil. For the acquisition of nutrients from the soil, plant root architecture changes, which results in the modulation of phytohormone in plant [5]. At all phenological phases, abiotic stressors inhibit plant growth and development, notably during the seed germination and reproductive growth stages. Among the majority of detrimental impacts generated by abiotic stressors on plants, nutritional shortage plays a crucial role (Table 1). Due to endogenous hormonal control or contact with root-zone bacteria that generate a range of hormones, plants resilient to abiotic stressors have a greater capacity for preferential nutrient absorption. Considering the significance of phytohormones in several physiologic processes of plants, exogenous administration of these hormones might increase plant stress tolerance. In addition, the application of bacteria that produce hormones may have diverse impacts in mitigating the negative effects of abiotic stressors on plants.

Under nutrient-deficient conditions such as low nitrogen (N), sulfur (S), and phosphorus (P), phytohormones play a key role in modulating lateral root development, thereby increasing the root surface area for enhanced nutrient uptake [31]. Specifically, under phosphorus (P) deficiency, both the number and length of root hairs are increased, which further improves the absorptive surface. P stress also induces a shift in root-to-shoot allocation, promoting greater root growth relative to shoot growth. Recent studies have suggested that under low-P conditions, elevated levels of phytohormones such as auxin, gibberellic acid, jasmonic acid, and salicylic acid contribute significantly to the remodeling of root system architecture [21].

### 3.2. Phytohormone-Mediated Transport and Assimilation of Nutrients

**Figure 2:**
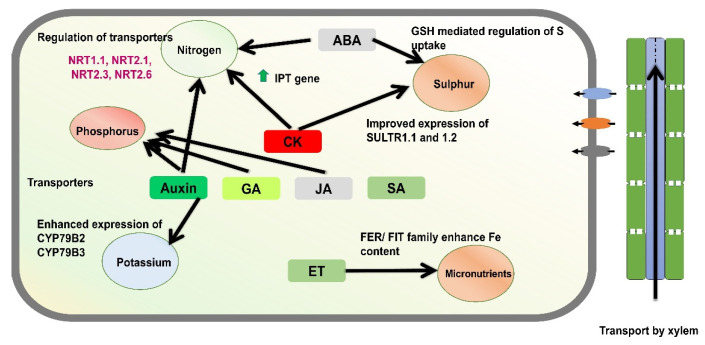
A schematic model illustrates the role of phytohormones in regulating nutrient acquisition in plants. Cytokinin plays a central role in the uptake of nitrogen and sulphur, with downstream components of cytokinin signaling influencing the expression of nitrogen transporters. Likewise, other phytohormones, including auxin, gibberellin (GA), and jasmonic acid (JA), contribute to the uptake and acquisition of phosphorus and potassium. In addition, the gaseous hormone ethylene (ET) has been implicated in the regulation of micronutrient acquisition, particularly under stress conditions.

The phytohormone-mediated transport and assimilation of nutrient crosstalk is a highly regulated network [79,80]. The synthesis and signalling of phytohormones such as cytokinin are regulated by the nutrient status, including NO_3_^−^ ions [81]. Nitrate levels in the plant significantly enhance the *IPT* gene, which results in the synthesis of cytokinin. Cytokinin stimulates the activity of nitrate reductase, which modulates the activity of nitrate transporters. Reports have suggested that nitrate transporters such as *NRT1.1*, *NRT2.1*, *NRT2.3*, and *NRT2.6* are interactively controlled by auxin, cytokinins, and ABA [82]. Other phytohormones, such as ABA, regulate plant growth and adaptability by modulating nutrients. Reports have suggested that ABA regulates the transport of several ABC (ATP-binding cassette) transporters under nitrogen stress conditions [71]. As discussed in the earlier section, the NPF transporter is involved mainly in the transport of nitrate ions. However, reports have also suggested that it is involved in the ABA absorption or ABA import transporter (AIT). *AtNPF4.6*/*AtNRT1.2,* also known as AIT1, facilitates the cellular absorption of ABA, which acts as an AMA importer in the process of stomatal aperture in the shoot part and, most importantly, under water stress conditions [17]. Therefore, it is probable that this transporter contributes to the drought response. Since the NPF family transports nitrate, further research is required to determine if nitrate signaling/nutrition interacts with ABA or stress tolerance (Figure 2).

As discussed in the earlier section, the physiological and molecular features of plant responses to variation in nutrient availability have been discussed and widely researched. However, our understanding of how plants monitor internal and external nutrient levels and homeostasis should be further investigated. The long-distance transport of nitrogen in plants was reported to be enhanced by cytokinin content, which also regulates the nitrogen status [83]. In combination with other types of signalling molecules, such as C-TERMINALLY ENCODED PEPTIDE DOWNSTREAMs, cytokinins participate in the control of nutrient acquisition and root system development during shoot-to-root communication [83]. In this regard, it has been proven that nitrate stimulates expression of the CK biosynthetic genes IPT3 (ISOPENTENYLTRANSFERASE 3) and CYP735A (encoding a cytochrome P450 monooxygenase), resulting in a greater build-up of CKs [84]. Reports suggested that CKs are known to negatively affect Pi starvation responses in *Arabidopsis* roots, such as the development of lateral roots and expression of Pi-deprivation-inducible genes, viz., *AtPT1* and *AtIPS1*. These aforementioned genes encode a high-affinity Pi transporter and a riboregulator, respectively [85]. Other reports also revealed that CK is involved in sulphur assimilation. Exogenous application of CK has been shown to cause enhanced expression of high-affinity sulphate transporters *SULTR1;1* and *SULTR1;2,* which leads to accumulation of S under low-S conditions [86].

The increase in the activation of genes responsible for encoding iron transporters, ferric reductase, and H^+^ATPase leads to an increase in root hair growth and organic acid secretion [87]. FER/FIT proteins, which are basic Helix–Loop–Helix (bHLH) transcription factors conserved in tomato and *Arabidopsis* and whose expression is also enhanced under iron-limiting circumstances, control gene expression in response to iron deprivation [88]. ABA is also known to affect sulphur homeostasis by increasing glutathione (GSH), a sulphur metabolism intermediary that regulates the redox status of plants. Therefore, ABA-mediated modulation of ROS levels has been invoked to explain its involvement in protecting plants against oxidative conditions caused by a range of stress situations, such as nutrient deficiencies [89].

Reports have demonstrated that the crosstalk and interactions of auxin and signalling pathways correlate with macronutrients such as nitrogen, phosphorus, and potassium [22]. Low-nitrate-cultivated soybean and *Arabidopsis* plants collected more auxins in their roots than plants grown in high-nitrate environments. In contrast, auxin concentrations in the shoots of nitrate-deprived plants were much lower than those of plants grown in a nitrate-rich media [16]. The absence of an influence of auxins on the low-sulfur-induced expression of *SULTR1;2* and APR2 suggests that these hormones control just a subset of plant responses to sulphur deprivation. Potassium status may also affect auxin accumulation, as indicated by the reduced expression of genes controlling auxin biosynthesis, such as CYP79B2 and CYP79B3, in potassium-depleted Arabidopsis plants that were subsequently supplied with this nutrient [14]. To demonstrate this regulatory system in its entirety, it is essential to examine the effect of ethylene on the expression of sensitive genes in plants with altered FER/FIT function. Coordinated regulation by ethylene of iron-responsive genes suggests likely control of the activity of FER/FIT family members, as indicated by the fact that transcription of FER/FIT genes, which stimulate gene expression in response to low iron availability, was also elevated under ethylene circumstances [90]. ABA affects sulphur homeostasis by increasing glutathione (GSH), a sulphur metabolism intermediary that regulates the redox status of plants. This ABA-mediated modulation of ROS levels is invoked to explain its role in protecting plants against oxidative conditions caused by nutrient deficiencies [91]. The regulation of iron-responsive genes by ethylene is suggested to involve the control of the activity of *FER/FIT* family members. Specifically, transcription of *FER/FIT* genes, which stimulate gene expression in response to low iron availability, was also elevated under ethylene circumstances. *FER/FIT* proteins are basic Helix–Loop–Helix (bHLH) transcription factors that control gene expression in response to iron deprivation [92].

## 4. Future Perspectives and Applications

The future application of phytohormones and nutrients may help translate the precise molecular insights from phytohormone–nutrient crosstalk into practical strategies for crop improvement, particularly through breeding programs and genetic engineering. One of the most promising avenues involves the direct manipulation of nutrient transporters and crucial regulatory factors to enhance acquisition and remobilization efficiency. For instance, successfully engineering high-affinity nitrogen (N) acquisition can be achieved by targeting transporters; studies have demonstrated that constitutive overexpression of high-affinity nitrate transporters like *OsNRT2.3b* and *OsNRT1.1b* increased N uptake and grain yield in rice [93]. For phosphorus (P) acquisition, incorporating quantitative trait loci (QTLs) like PSTOL1 (phosphorus starvation tolerance 1) into sensitive varieties is a proven strategy, as this protein kinase enhances root growth and Pi uptake even under low-Pi conditions, leading to significantly better yields [11]. Furthermore, adjusting regulatory networks is essential, such as utilizing the RING E3 ubiquitin ligase NLA (Nitrogen Limitation Adaptation), which controls the degradation of plasma membrane-localized phosphate transporters (PHT1/PHT2) and regulates nitrate remobilization (NRT1.7) as a molecular target to fine-tune nutrient homeostasis during stress. Finally, the comprehensive genetic polymorphism data now available enable the identification of desirable alleles, supporting haplotype-based breeding to customize high-quality crops with enhanced nutritional value.

Another critical area for future work lies in integrating advanced technologies to inform agronomic management and environmental resilience. Integrating sophisticated tools, such as omics approaches (transcriptomics, metabolomics, and phenomics), provides the necessary depth to unravel complex nutrient–stress interactions and identify novel targets for crop improvement. Modern genome editing tools, such as CRISPR-Cas technology, can be deployed with high-throughput sequencing to optimize inorganic and organic nitrogen transport systems, considering their temporal expression and localization, thereby fine-tuning N uptake, metabolism, and whole-plant partitioning. In terms of field management, this understanding enables the development of precision agriculture: for example, the concept of “smart plants” harbouring reporter genes sensitive to nutrient starvation signals can allow real-time monitoring of plant nutritional status, facilitating precision management of fertilization to reduce chemical inputs while sustaining yield. Complementary approaches include the exogenous application of phytohormones or hormone-producing bacteria to directly enhance plant stress tolerance and nutrient absorption capacity directly, addressing nutritional imbalances under adverse conditions. This focus on system integration and precision targeting is fundamental to developing sustainable agricultural systems that maintain productivity in the face of climate challenges.

## 5. Conclusions

Plants have evolved sophisticated adaptive mechanisms to cope with nutrient deficiencies. A primary strategy involves modifying their root system architecture (RSA), adjusting branching patterns and overall root structure to enhance nutrient uptake, especially when nutrients are scarce or have limited solubility. Crucial to this process are membrane transporter proteins, including the nitrate (NRT), ammonium (AMT), phosphate (PHT), and potassium (HAK) families, which mediate nutrient uptake and redistribution under stress conditions. These transporters undergo specific transcriptional and post-transcriptional regulation, modulating their activity in response to external nutrient availability. Phytohormones play a pivotal role in orchestrating plant responses to nutrient-deficient conditions and remodelling root architecture. To improve nutrient use efficiency and enhance crop resilience to abiotic stresses, several strategies are proposed. These include the overexpression of key transporters, genetic modifications targeting stress-responsive pathways, and the application of exogenous phytohormones. Moreover, integrating omics approaches such as transcriptomics, metabolomics, and phenomics is essential for provide deeper insights into nutrient–stress interactions and identify promising targets for crop improvement. By unravelling these complex regulatory networks, this research aims to pave the way for sustainable agricultural practices, ensuring productivity in the face of escalating climate change.

## Figures and Tables

**Table 1:** Genes and molecular mechanisms regulating phytohormone responses, abiotic stress adaptation, and physiological functions in plants.

Gene Name	Hormone Regulating/Involved	Abiotic Stress Condition(s)	Physiological Function/Role	Reference
AtABCG25 (At1g71960)	Abscisic acid (ABA)	Environmental stresses, Abiotic stress responses	ATP-binding cassette (ABC) transporter that functions as an ABA exporter and involved in the intercellular ABA signaling pathway.	[71]
AMT1AtAMT1;1, AtAMT1;2, AtAMT1;3, AtAMT2;1	None explicitly (regulated by root glutamine levels/N demand)	Nitrogen (N) deficiency	Ammonium transporters mediating root ammonium fluxes in response to cellular or whole-plant demand for nitrogen.	[56,72]
AtHAK5	Jasmonic acid (JA) (JA-linked response observed in plate-grown plants on low-K agar)	Potassium (K^+^) starvation/deprivation	Potassium transporter (KUP/HAK/KT family) that takes part in K^+^ deprivation-induced high-affinity K^+^ uptake in roots.	[51]
AHK1, AHK2, AHK3, CRE1 (AHK4)	Cytokinin (CK), ABA	Drought, salt stress, ABA responses	Stress-responsive non-ethylene histidine kinases (HKs) that function as cytokinin receptors and play roles in the regulation of plant responses to abiotic stresses. CRE1 mutation impairs cytokinin-induced repression of phosphate starvation responses.	[73]
ABI5, AREB/ABFs	ABA	Dehydration response, osmotic stress	bZIP transcription factors (TFs) mediating ABA-regulated gene expression during dehydration response in vegetative tissues and seed maturation.	[74]
COI1 (Coronatine-Insensitive 1)	Jasmonic acid (JA)	K deficiency (low K)	F-box protein acting as a JA receptor. Targets transcriptional repressors for degradation. Mediates transcriptional responses of *Arabidopsis thaliana* to external potassium supply.	[14]
LOX2 (At3g45140)	JA	K deficiency	Encodes a 13 (S)-lipoxygenase (LOX), which catalyzes the initial step of JA production/biosynthesis.	[14]
AOS, AOC1, OPR3	JA (oxylipin biosynthetic pathway)	K deficiency	Biosynthetic enzymes involved in the oxylipin production pathway downstream of LOX2.	[14]
VSP2 (At5g24770)	JA	K deficiency	Vegetative storage protein, well-known target of JA-signaling. It is an important nitrogen (N) store and plays a role in plant defense against pests.	[14,75]
CYP79B2, CYP79B3	JA (induction requires intact COI1 signaling)	K starvation (K deficiency)	Catalyze the first step in the biosynthesis of tryptophan-derived indole glucosinolates (GLS).	[76,77]
CYP79F1, CYP79F2	None explicitly	K deficiency	Encode enzymes that catalyze the synthesis of methionine-derived aliphatic GLS.	[78]
BnaPHT1s (Phosphate Transporter Family 1 members in *B. napus*)	Auxin (IAA), cytokinin (CTK)	Phosphorus (P) shortage/deprivation, nitrogen (N), potassium (K), sulphur (S), iron (Fe) nutritional stressors, salt, drought	Phosphate transporters involved in Pi acquisition and homeostasis. Involved in crosstalk for sensing external nutrient status and environmental stresses.	[50]
OsPT8 (OsPht1;8)	Auxin	Phosphate (Pi) deficiency/starvation	Pi transporter involved in Pi uptake, translocation from root to shoot, and Pi homeostasis in rice. Its expression is induced by auxin and P starvation.	[64]
PpeKUP genes (KT/HAK/KUP family in peach)	None explicitly	K^+^ deficiency, K^+^ excess, PEG (drought stress), Pb (heavy metal), Cd (heavy metal), Al	Potassium transporters facilitating K^+^ uptake and transport and maintaining K^+^ homeostasis in peach seedlings.	[52]
HvHAKs (HAK/KUP/KT family in barley)	Abscisic acid (ABA), methyl jasmonate (MeJA)	Salt stress, hyperosmotic stress (drought/PEG8000), potassium (K) deficiency	K^+^ uptake and transporters involved in maintaining K^+^ Na^+^ homeostasis and salt tolerance and enhancing intracellular osmotic adjustment/drought tolerance.	[13,21]
TIR1, ARF19	Auxin	Low phosphorus (LP)	Auxin signaling components involved in the LP response.	[21]
ACCO	Ethylene (biosynthesis pathway)	Low phosphorus (LP)	Involved in ethylene biosynthesis pathway.	[21]
MtNPF6.8 (MtNRT1.3)	IAA (Auxin), ABA	Nitrate (N) regulation/N-free medium (N starvation)	Nitrate transporter, also able to transport ABA or facilitate ABA transport in a heterologous system. Proposed role in mediating nitrate regulatory effect on lateral root (LR) development.	[17]
SlAMT1-1, SlAMT1-2, SlAMT1-3 (ammonium transporters in tomato)	ABA	Drought, salt stresses	Plasma membrane proteins that function in ammonium transmembrane transport. Also involved in lateral root formation/branching and response to ABA.	[44,69]
OsGS1;1, OsGS1;2 (glutamine synthetase)	-	Salt, drought, and cold stress	Key enzyme in nitrogen metabolism. Catalyzes the incorporation of inorganic ammonium into glutamine. Overexpression modifies N metabolism and abiotic stress responses.	[69]

## Data Availability

All materials and datasets related to this publication are accessible to the readers.
